# Influential Factors in Adherence to the Therapeutic Regime in Hypertension and Diabetes

**DOI:** 10.17533/udea.iee.v37n3e02

**Published:** 2019-10-23

**Authors:** Dora Inés Parra, Sandra Lucrecia Romero Guevara, Lyda Z. Rojas

**Affiliations:** 1 Nurse, Masters. Professor, Universidad Industrial de Santander, Bucaramanga, Colombia. Email: doiparra@uis.edu.co Universidad Industrial de Santander Universidad Industrial de Santander Bucaramanga Colombia doiparra@uis.edu.co; 2 Nurse, Masters. Professor, Universidad Industrial de Santander, Bucaramanga, Colombia. Email: salurome@uis.edu.co Universidad Industrial de Santander Universidad Industrial de Santander Bucaramanga Colombia salurome@uis.edu.co; 3 Nurse, PhD. Professor, Universidad Industrial de Santander, Bucaramanga, Colombia. Email: lyda.rojas@correo.uis.edu.co Universidad Industrial de Santander Universidad Industrial de Santander Bucaramanga Colombia lyda.rojas@correo.uis.edu.co

**Keywords:** treatment adherence and compliance, hypertension, diabetes mellitus, type 2, risk factors, cross-sectional studies., cooperação e adesão ao tratamento, hipertensão, diabetes mellitus tipo 2, fatores de risco, estudos transversais.

## Abstract

**Objective.:**

To determine the factors associated with adherence to the therapeutic regime in patients with hypertension and type 2 diabetes mellitus cared for in primary care centers.

**Methods.:**

This was an analytical cross-sectional study, conducted with 500 patients from two institutions in Bucaramanga (Colombia). Adherence to the therapeutic regime was measured with the label of Nursing outcomes “Treatment Behavior: Illness or Injury” and the instrument “Factors that influence on adherence to pharmacological and non-pharmacological treatments” by Ortiz Suárez was used.

**Results.:**

Factors affecting negatively adherence to the therapeutic regime were: belonging to the subsidized regime, never being able to read written information about the management of their disease, and never receiving information about benefits of the medications ordered by the physician. On the contrary, positive influence was noted by referring “never” to the following statements, which impacted positively on adherence: the diverse occupations you have in and out of the house hinder your following the treatment; when your symptoms improve, do you interrupt the treatment? previously, have you had difficulties in complying with your treatment and believe there are difficult-to-change customs about foods and exercises?

**Conclusion.:**

Two socioeconomic factors and one related with the health system and staff affected negatively on adherence to the therapeutic regime, while four factors related with the therapy favored it. The factors identified could be used in the design of nursing interventions to improve adherence in this population.

## Resumo

**Objetivo.:**

Determinar os fatores associados à aderência ao regime terapêutico em pacientes com hipertensão e diabetes mellitus tipo 2 atendidos nos centros de atenção primária.

**Métodos.:**

Estudo de corte transversal analítico, realizado em 500 pacientes de duas instituições de Bucaramanga (Colômbia). A aderência ao regime terapêutico foi medida com a etiqueta de Resultados de Enfermagem “Conduta terapêutica: doença ou lesão” e se empregou o instrumento “Fatores que influem na aderência a tratamentos farmacológicos e não farmacológicos” de Ortiz Suárez.

**Resultados.:**

Os fatores que afetaram negativamente a aderência ao regime terapêutico foram: pertencer ao regime subsidiado, nunca poder ler a informação escrita sobre o manejo da sua doença e nunca receber informação sobre benefícios dos medicamentos ordenados pelo médico. Pelo contrário, influíram positivamente referir “nunca” às seguintes afirmações, impacto positivamente a aderência: as diversidades ocupações que tem dentro e fora do lar lhe dificultam seguir o tratamento; quando melhoram seus sintomas, você suspende o tratamento; anteriormente há apresentado dificuldades para cumprir seu tratamento e acredita que há costumes sobre alimentos e exercícios difíceis de mudar.

**Conclusão.:**

Dois fatores socioeconômicos e um relacionado com o sistema e equipamento de saúde, afetaram negativamente a aderência ao regime terapêutico, enquanto que quatro fatores relacionados com a terapia a favoreceram. Os fatores identificados poderiam ser utilizados no desenho de intervenções de enfermagem para melhorar a aderência nesta população.

## Introduction

The concept of therapeutic adherence was defined by the World Health Organization with a more inclusive vision, which highlights the diverse interdisciplinary recommendations that must be complied by patients with chronic disease and, additionally, evidences the patient’s active participation during the disease process.(1) In this sense, it was agreed that therapeutic adherence corresponded to the “degree to which a person’s behavior in relation with taking medications, following a dietary regime, or modifying life habits corresponds to recommendations agreed by a health care provider”.(1) The low level of adherence to the therapeutic regime is well-known throughout the spectrum of chronic disease.(2) In developed countries, only 50% of the people who suffer from chronic disease comply with the prescribed treatment, while in developing countries this adherence may be lower because of the scarcity of resources and inequities in access to health services.(1,3) 

Lack of adherence is attributed to multiple factors; according to the World Health Organization, five dimensions exist that constitute the Multidimensional Model of Adherence: health system and staff, therapy, disease, patient, and socioeconomic aspects.(1) Regarding arterial hypertension and type 2 diabetes mellitus (T2DM), non-adherence to the therapeutic regime constitutes a public health problem. In this respect, very variable figures have been reported of the prevalence of adherence to medications ranging between 24.1% and 92.7%(4) in patients with arterial hypertension and between 38.5% and 93.1%(5) in patients with T2DM. With non-pharmacological aspects, non-adherence is most common, thus, non-compliance frequencies have been found ranging between 33.2% and 25% in diabetic patients(6,7) and 68.8% in hypertensive patients,(8) while for dietary concerns, non-compliance has been reported between 88% and 44.8% in diabetic patients(6,7) and 30.9% in hypertensive patients.(8) 

Bearing in mind the disease burden In arterial hypertension and T2DM and the low therapeutic adherence (on average 50%),(1) in the future it is expected that complications from these two pathologies to become the principal threats to public health resources globally(9) because this implies higher rates of hospitalization and, hence, increased economic and social costs. Research has been conducted to identify the factors associated to adherence to treatment,(10) however, most has focused on the aspect of pharmacological compliance, without measuring adherence to changes in lifestyle, like diet, physical activity, consumption of alcohol and tobacco, among others, of which evidence exists that its adhesion contributes to achieving the therapeutic objectives.(11,12) In this sense, the literature has indicated that nursing, as part of the health staff, plays a fundamental role in improving this problem, especially through education actions aimed at modifying lifestyles and understanding the disease.(13) Consequently, the aim of this study was to determine the factors associated with adherence to the therapeutic regime (pharmacological and non-pharmacological treatment) in patients with arterial hypertension and T2DM cared for in primary care centers in the city of Bucaramanga in Colombia.

## Methods

*Study design and population.* An analytical cross-sectional study was conducted in 2013 in patients with medical diagnosis of arterial hypertension or T2DM belonging to cardiovascular risk programs from two primary care institutions, one of public nature from the city of Bucaramanga-Colombia. The inclusion criteria involved individuals over 18 years of age, totally independent of their care and actively attending cardiovascular risk programs. The study excluded pregnant women, individuals with altered mental sphere, with chronic or severe alterations, and with communication limitations. To calculate the sample size, the study took as reference an available population of 7,000 people, an expected adherence to the therapeutic regime of 50%, 95% confidence interval, a design effect of 1, and losses due to non-response rate of 20%, obtaining as a result a sample size of 438 people. Probabilistic sampling (simple random) was used for sample selection. 

*Measurements.* The work assessed sociodemographic and clinical factors, like age, sex, marital status, socioeconomic level, distance between the place of residence and the health care center, social security regime, occupation, disease suffered, time registered with the cardiovascular risk program, number of hospitalizations within the last year, short-form Charlson Comorbidity Index,(14) risk of general cardiovascular disease at 10 years, according to Framingham,(15) and number of pills taken per day. This information was gathered by reviewing the participants’ medical records and confirmed during the application of self-report instruments. Adherence to therapeutic regime was measured with the label Nursing Outcomes Classification “Treatment Behavior: Illness or Injury”,(16) which evaluates compliance with the medication regime, diet and activity prescribed, supervision of the therapeutic effects and avoidance of behaviors that potentiate the pathology. This is a self-report instrument with dichotomy response scale (yes/no), comprised of 13 items grouped into five dimensions. Total scores vary from 0 to 13 points, indicating that higher scores mean greater adherence and vice versa. The construct validity and test-retest reproducibility of said instrument yielded as a result a total explained variance of 67.62%, Cronbach’s alpha internal consistency of 0.60, and a test-retest reproducibility of 0.63. Factors related with adherence were measured with the instrument “Factors that influence on adherence to pharmacological and non-pharmacological treatments”,(17) which evaluates factors related with the following aspects: (I) socioeconomic; (II) provider: health system and staff; (III) therapy, and (IV) patient. This instrument can be self-completed or completed with aid; it has a Likert-type response scale with three options (never / sometimes / always) and it is comprised of 24 items grouped into four dimensions that explain 45% of the variance and report a Cronbach’s alpha of 0.60.

*Statistical analysis.* The total score of adherence to the therapeutic regime did not show normal distribution (Shapiro-Wilk test, *p* <0.001); thereafter, it was described with the median, first and third quartile. A bivariate analysis was performed between the total score of adherence to treatment (quadratic transformation) and the sociodemographic and clinical variables and factors influencing adherence to the therapeutic regime through linear regressions. A multivariate analysis was carried out, an automated stepwise backward regression methodology was used; the variables obtaining a value of *p* ≤0.20 in the bivariate analysis were included in the multiple linear model, variables with *p* ≤0.05 were retained in the model. No variable was considered as the main exposure. The original scale of the influential factors instrument has three response options (never/ sometimes / always); for this analysis, the categories sometimes and always were grouped as reference category and never as risk category. Once the model was determined, the multi-collinearity was evaluated with thee variance inflation factor (VIF), considering collinearity with VIF>10. Model specification was evaluated with *linktest*, indicating adequate specification with non-significant hatsq (*p*≥0.05). The assumptions of the linear model were assessed: linearity was evaluated with studentized residuals, the normality of the residuals was explored with the Q-Q-plot graphic, P-P plot and the Shapiro Wilk test; homoscedasticity with the Cook-Weisberg test, fulfilling all the assumptions of the linear model. 

*Ethical considerations.* This study was approved by an institutional research ethics committee and was approved by the participating institutions. The research subjects provided a previously written informed consent. It also received written authorization by the author of the instrument “influential factors in adherence to pharmacological and non-pharmacological treatments” for use in this research.

## Results

In all, 500 patients were studied (69.4% had arterial hypertension, 9.0% DM, and 21.6% had both pathologies). The median age of the population was 68 years of age (Q1=59 years; Q3=75) and 69.0% were women; 52.2% were married/common law; 58% were in the low socioeconomic level; 65% had primary education; 44.8% were housekeepers. The median of pills taken by patients per day was five pills (Q1=3; Q3=7 pills). Lack of comorbidity was identified in 90.0%, according to the Charlson index 12% and 48.47% had high and very high risk of cardiovascular disease at 10 years, according to Framingham. 

The median of the total score of adherence to the therapeutic regime was 10 over a total of 13 points (Q1=9; Q3=11) (total score of adherence with quadratic transformation, mean of 99.5±37.5 points). The bivariate analysis with sociodemographic and clinical factors shows that having a low socioeconomic level and being in the subsidized health regime diminish the adherence score, while a greater time being registered in the cardiovascular risk program increases the level of adherence (Table 1).

**Table 1:** Sociodemographic and clinical factors of adherence to the therapeutic regime. Bivariate analysis (*n*=500)

Adherence to the therapeutic regime	β	95% CI	p value
Age (years)	0.02	-0.27; 0.31	0.889
Sex			
Female	Reference		
Male	4.14	-2.98; 11.27	0.254
Marital status			
Single	Reference		
Married	8.14	-1.36; 17.65	0.093
Common law	-0.90	-13.22; 11.42	0.886
Divorced/Separated	-8.84	-23.74; 6.06	0.244
Widowed	3.36	-7.04; 13.77	0.525
Socioeconomic level			
Medium/high	Reference		
Low	-7.38	-14.04; -0.72	0.030
Distance from place of residence/care center (minutes)	0.07	-0.12; 0.26	0.473
Social security regime			
Contributive	Reference		
Subsidized	-16.29	-22.74; -9.84	0.001
Occupation			
Employed	Reference		
Independent	-5.32	-20.61; 9.97	0.495
Unemployed/laid off	-2.68	-18.01; 12.64	0.731
Pensioned/Retired	11.38	-3.43; 26.20	0.132
Home/Housekeeper	-1.96	-15.61; 11.68	0.778
Disease suffered			
Hypertension	Reference		
Diabetes mellitus	-9.55	-21.23; 2.12	0.109
Hypertension and diabetes mellitus	-0.59	-8.71; 7.53	0.886
Time registered in the cardiovascular risk program (months)	0.28	0.10; 0.47	0.003
Number of hospitalizations in the last year (times)	-1.27	-5.50; 2.96	0.556
Charlson index (Continuous)	-1.27	-5.85; 3.31	0.586
Risk of general cardiovascular disease at 10 years (Framingham)	-0.13	-0.50; 0.23	0.458
Number of pills/day	0.86	-0.47; 2.19	0.206

*The total score of adherence was used with a quadratic transformation.

The socioeconomic-type influential factors that do not favor adherence include not having the economic resources to travel to the consultation site, not being able to read written information about managing their disease, and not having support from their families or close friends to comply with their treatment; on the other hand, when people reported that changes in diet were not made difficult due to the high cost of foods recommended, this was associated with increased adherence. Regarding factors related with the health system and staff, when patients stated that the people caring for them never respond to their concerns and difficulties with respect to their treatment; this was associated with a decreased level of adherence. With regards to the influential factors related with the therapy, it was evident that the majority of the factors affect positively on adherence, and on factors related with the patient; people who never manifest interest for knowing about their health condition and how to care for themselves had lower adherence (Table 2).

**Table 2:** Influential factors (socioeconomic, provider, therapy and patient) in adherence to the therapeutic regime. Bivariate analysis (*n*=500)

Adherence to the therapeutic regime	β	95% CI	***p*-value**
Socioeconomic			
Family has economic availability to meet basic needs (diet, health, housing, education).	-4.79	-24.14; 14.55	0.626
Can afford medications.	3.03	-4.48; 10.56	0.428
Has economic resources to travel to the consultation.	-10.32	-20.09; -0.54	0.039
Changes in the diet are difficult due to the high cost of the foods recommended.	8.47	1.78; 15.16	0.013
Can read written information on managing their disease.	-14.63	-21.50; -7.76	0.001
Have support from their families or close acquaintances to comply with their treatment.	-11.16	-19.39; -2.93	0.008
Provider: Health system and staff			
People caring for them respond to their concerns and difficulties with respect to their treatment.	-20.61	-39.28; -1.94	0.031
Are aware that their physicians control if they are following the treatment by the questions they make.	-12.20	-25.45; 1.05	0.071
Receives information about the benefits of the medications ordered by their physicians.	-5.40	-12.80; 2.00	0.152
Receives guidance on how to adjust schedules to the medications according to their daily activities.	-0.17	-9.99; 9.64	0.972
In case you failed in your treatment, would your physician and nurse understand your motives?	-5.70	-12.80; 1.39	0.115
The physician and nurse give you explanations with words you and your family understand.	-15.63	-33.30; 2.03	0.083
The physician and nurse have explained to you what results the treatment you are getting will have on your health.	0.46	-7.17; 8.11	0.905
Do you feel the physician and you coincide in the hope of improving with the treatment and the changes being made in your habits.	-7.21	-21.10; 6.66	0.308
The therapy			
The diverse occupations you have in and out of the house make it difficult to follow the treatment.	32.09	18.69; 45.48	0.001
The distances from your house or work to the consultations hinder compliance with the appointments.	6.53	-2.27; 15.34	0.146
Have doubts about how to take their medications, regarding the amount, schedules, and the relationship with foods.	15.83	4.71; 26.95	0.005
When your symptoms improve, do you interrupt the treatment?	20.81	10.15; 31.47	<0.001
Have you previously had difficulties to comply with your treatment?	21.22	10.66; 31.78	<0.001
Do you believe there are customs about foods and exercise that are difficult to change?	10.87	4.00; 17.75	0.002
The patients			
Are convinced the treatment is beneficial and that is why they continue with it.	-31.01	-67.98; 5.94	0.100
Are interested in knowing about their health condition and how to care for themselves.	-48.81	-91.35; -6.27	0.025
Believe it is important to follow their treatment to improve their health.	-22.95	-59.96; 14.05	0.224

The multivariate analysis found seven factors associated with adherence to the therapeutic regime; three of which affect negatively on adherence, two of them related with socioeconomic aspects: *belonging to the government subsidized regime* compared to the so-called contributive (private) regime and *never being able to read written information about managing their disease*; the third was related with the health system and staff: *not receiving information about the benefits of the medications ordered by the physician*. Finally, when patients state “never” to each of the following four statements, adherence is favored positively: *the diverse occupations in and out of the house hinder their following the treatment*; *when your symptoms improve, do you interrupt treatment*; *have you previously had difficulties to comply with your treatment*; and *do you believe there are customs about foods and exercise that are difficult to change* (Table 3). 

**Table 3:** Influential factors (socioeconomic, provider, therapy, and patients) on adherence to the therapeutic regime. Multivariate analysis (*n* = 500)

Adherence to the therapeutic regime	β	95% CI	***p*-value**
Socioeconomic			
Social security (Subsidized vs. Contributive)	-15.43	-22.83; -8.03	<0.001
Can read written information about managing their disease	-8.16	-15.56; -0.76	0.031
Provider: Health system and staff			
Receives information about the benefits of the medications ordered by their physician	-8.72	-16.15; -1.29	0.022
The therapy			
The diverse occupations in and out of the house hinder their following the treatment	17.36	3.92; 30.79	0.011
When your symptoms improve, do you interrupt the treatment?	12.64	2.34; 22.94	0.016
Have you previously had difficulties to comply with your treatment?	19.52	8.86; 30.18	<0.001
Do you believe there are customs about foods and exercise that are difficult to change?	7.26	0.73; 13.80	0.029

## Discussion

Non-adherence is a global phenomenon of serious consequences and it is present in almost every chronic disease. It is related with multiple aspects, among them socioeconomic, the health system and staff, therapy, the disease, and patients, which makes it complex to framework and intervene.(1) The principal findings of the study permit showing that factors related specifically with therapy affect the total score of adherence to the therapeutic regime, among them aspects concerning diverse occupations, antecedents of difficulties, interruption of treatment, and beliefs about eating habits and exercise; followed by socioeconomic factors and system factors, like the social security regime, not being able to read information about the disease, and not receiving information about the benefits of the medications ordered by the health providers. 

This study showed that not being able to read written information about managing the disease and not receiving information about the benefits of the medications ordered by the physician were associated with low levels of adherence; these two aspects are highly related with the level of schooling and knowledge. For this study, the population was principally characterized by having a low (58.0%) and medium (41.4%) socioeconomic level, likewise, 11.0% was illiterate and 65.4% only had primary school education. These results agree with the findings described by Castaño-Castrillón *et al*.,(18) who, with 200 hypertensive patients cared for in different low-complexity healthcare centers in the city of Manizales - Colombia, measured adherence with the questionnaire by Martín-Bayarre-Grau (MBG) and found low levels of adherence associated with low educational level and low knowledge of the pathology. Also, Ghembaza *et al.,* (19) found a positive relationship between patients’ knowledge about the complications of hypertension and adherence (*p*<0.003; OR=0.46) in a study conducted with 453 patients from a public primary care center in the department of Tlemcen in Algeria. Likewise, Rodríguez-Abt *et al*.,(20) in 2017, investigated the association between knowledge about hypertension and adherence to the treatment (MBG measure) in 340 hypertensive patients cared for in cardiology consultation in a National Hospital in Lima - Peru, and determined that patients with low knowledge of hypertension have 1.5 times more probability of having partial adherence to the treatment than patients with high knowledge. Multiple studies have evidenced the relationship between the level of knowledge and adherence to the treatment in both hypertension and diabetes mellitus.(7,21)

With respect to improving symptoms and interrupting the treatment, it was found that not interrupting the treatment, even if symptoms improve, favors adherence to the treatment. In this sense, a systematic review of qualitative research on the perspectives of hypertension and adherence to medications, which synthesized information from 59 articles (53 of them qualitative) and studies derived from 16 countries, evidenced that participants reduce or interrupt intentionally the treatment without consulting their treating physician; commonly, participants perceive that their blood pressure improves when symptoms diminish or when they are not stressed, and that the treatment is not necessary at that those moments.(22)

In addition, it was found that belonging to the subsidized regime is associated negatively with adherence to treatment, which may be because people belonging to this regime are of scarce economic resources, which hinders compliance with the therapeutic regime, for example, adoption of an appropriate diet according to their health condition, frequent follow-up visits with the treating physician, and the need to purchase medications when these are not readily dispensed by the health provider. In this respect, the scientific literature indicates that lack of economic resources do not favor adherence, given that, for example, patients stop taking medications due to their high costs and stop attending their appointments, among others.(5,7,21,22) 

Other relevant aspects were related with stating “never” having had prior difficulties to comply with the treatment, or diverse occupations in and out of the house that hinder their following the treatment, which was associated positively with adherence. In that respect, some studies have reported that being busy keeps patients from taking their medications, engaging in exercise, or attending control appointments.(7,22) Likewise, it was found that if they never “believe that customs exist about foods and exercise that are difficult to change”, this favors adherence positively. In this regard, it has been documented that the attitudes of the individuals, their sex, lack of motivation, beliefs, being busy, and the sense of self-care, among others, influence adherence significantly and, hence, on the adoption of healthy behavioral changes.(7, 23,24)

Among the strengths of this study, we highlight the measurement of adherence to treatment, pharmacological and non-pharmacological, in patients with arterial hypertension and T2DM. Most studies only evaluate adherence to pharmacological treatment. Similarly, the study highlights the size and probabilistic sample selection, which makes it representative and control selection bias. Further, the influential factors of adherence were studied framed within a model scientifically known and established by the World Health Organization (Multidimensional Model of Adherence). However, some limitations to be noted emerge, having considered only patients who were active in cardiovascular risk programs, which could represent a population with higher adherence with respect to those who are not active. In addition, as known, self-report instruments are easy and practical to use and some have been validated against objective measurement methods in elderly populations with hypertension and Parkinson’s disease, nevertheless, they have the disadvantage of overestimating the measurement of adherence and it is likely that each method varies in its performance, according to the characteristics of the population studied.(10)

Regarding the implications of this study for the practice, for health services providers and for health professionals, it is important to know the factors that influence on the behavior of adherence of individuals with processes of chronic disease, like arterial hypertension and T2DM, given that these affect people’s behaviors, leading them to not complying strictly with the therapeutic regime prescribed and, thereby, not complying with the therapeutic objectives. In this sense, nursing - as part of the health staff in primary care and within the current role highlighted by the Comprehensive Health Care Model in Colombia - can lead individual and collective actions to maintain health and diminish degrees of disability in people with these pathologies. Similarly, the influential factors identified in this study could be useful to design future studies of nursing intervention aimed at improving adherence to the therapeutic regime in this population, a key aspect in optimizing the use of resources, an action that is part of the responsibilities of nursing management. (25)

To conclude, this study identified the therapy, socioeconomic, and health system/health staff factors associated with measuring adherence to the therapeutic regime (pharmacological and non-pharmacological) in patients with arterial hypertension and T2DM; three of them affecting adherence negatively (subsidized social security regime, not being able to read written information about managing the disease, and not receiving information about the benefits of the medications ordered by the health providers) and four factors favoring adherence (not having diverse occupations, not interrupting treatment even if symptoms improve, not having antecedents of difficulties to comply with the treatment, and not believing that customs exist about foods and exercises that are difficult to change). The influential factors identified in this study could be useful to design future studies of nursing intervention aimed at improving adherence to the therapeutic regime in this population. 
